# Mechanical Behavior of Topology-Optimized Lattice Structures Fabricated by Additive Manufacturing

**DOI:** 10.3390/ma18153614

**Published:** 2025-07-31

**Authors:** Weidong Song, Litao Zhao, Junwei Liu, Shanshan Liu, Guoji Yu, Bin Qin, Lijun Xiao

**Affiliations:** 1 State Key Laboratory of Explosion Science and Safety Protection, Beijing Institute of Technology, Beijing 100081, China; swdgh@bit.edu.cn (W.S.); zlt960620@163.com (L.Z.); 2 Institute of Systems Engineering, China Academy of Engineering Physics, Mianyang 621999, China; liujunwei0409@163.com; 3 Inner Mongolia Metal Material Research Institute, Yantai 264003, China; yuguoji0317@163.com; 4 National Key Laboratory of Transient Impact, No. 208 Research Institute of China Ordnance Industries, Beijing 102200, China; altanana@sina.com

**Keywords:** lattice structure, topology optimization, additive manufacturing, maximum bulk modulus, elastic isotropy

## Abstract

Lattice-based metamaterials have attracted much attention due to their excellent mechanical properties. Nevertheless, designing lattice materials with desired properties is still challenging, as their mesoscopic topology is extremely complex. Herein, the bidirectional evolutionary structural optimization (BESO) method is adopted to design lattice structures with maximum bulk modulus and elastic isotropy. Various lattice configurations are generated by controlling the filter radius during the optimization processes. Afterwards, the optimized lattices are fabricated using Stereo Lithography Appearance (SLA) printing technology. Experiments and numerical simulations are conducted to reveal the mechanical behavior of the topology-optimized lattices under quasi-static compression, which are compared with the traditional octet-truss (OT) and body-centered cubic (BCC) lattice structures. The results demonstrate that the topology-optimized lattices exhibited superior mechanical properties, including modulus, yield strength, and specific energy absorption, over traditional OT and BCC lattices. Moreover, apart from the elastic modulus, the yield stress and post-yield stress of the topology-optimized lattice structures with elastically isotropic constraints also present lower dependence on the loading direction. Accordingly, the topology optimization method can be employed for designing novel lattice structures with high performance.

## 1. Introduction

With the rapid advancement of technology, engineering fields now demand superior mechanical properties from lattice structures. Compared to traditional structures, lattice structure has excellent characteristics such as lightweight [1,2,3], high specific stiffness [4,5,6], high specific strength [7,8,9], outstanding energy absorption performance [10,11,12], controllable Poisson’s ratio [13,14,15,16], and designable anisotropy [17,18,19,20]. Furthermore, the lattice structure can be adapted to meet high load-bearing requirements while retaining its structural characteristics. Consequently, it can be widely applied to aerospace, medicine, transportation, and some other fields [21,22,23].

Generally, traditional engineering materials are usually isotropic, such as polycrystalline metals, polymers, and ceramics [24,25,26,27], which do not need to consider directionality in practical applications [28]. Unlike traditional engineering materials, lattice structures exhibit different mechanical properties in different loading directions due to their geometric configuration. Therefore, the mechanical behavior of the lattice structure is significantly dependent on the loading directions. For instance, the stiffness of a simple cubic lattice structure in the [1, 0, 0] direction is significantly higher than that in [1, 1, 1] direction [29,30]. The isotropic property enables the lattice structure to effectively withstand external loads from any loading direction and avoid structural collapse caused by uneven loads. However, the anisotropic performance of the lattice structure severely limits its engineering application. As a result, it is necessary to evaluate the anisotropy degree of lattice structures [31,32,33].

Improving the mechanical properties of lattice structures is a continuous goal in their design. Numerous approaches exist for designing lattice structures [34,35,36]. The topology optimization method based on homogenization has been widely used in the engineering field to design lattice structures for optimal stiffness or minimal mass [37,38,39]. After decades of development, new topology optimization methods designed to achieve maximum stiffness under specified constraints are continually being proposed. Several topology optimization methods are widely used to design novel structures, including the penalty function-based isotropic solid material variable density method [40,41], the level set method [42,43,44], the BESO method [45,46], and the optimization of mobile deformable components [47,48]. Sigmund et al. [41] successfully applied topology optimization methods to design lattice structures with a negative Poisson’s ratio. Consequently, significant progress has been made in designing lattice structures with excellent mechanical properties through the use of topology optimization [49,50,51,52,53,54,55].

The topology optimization method can be employed not only to design structures with maximum stiffness but also to design structures with specific mechanical properties. The mechanical properties within a lattice structure cell can be obtained by the homogenization method according to its microstructure. Therefore, designing new configurations based on specific mechanical properties can be considered an optimization problem. Topology optimization can use various material properties as objective functions, such as stiffness [56], Poisson’s ratio [57], energy absorption [54], bulk modulus, shear modulus [58], and isotropic [59,60,61]. Zhao et al. [59] employed a combination of artificial neural network and elastic isotropy optimization to obtain the isotropic bamboo-inspired body-centered cubic lattice structure. Jiang et al. [60] proposed a novel class of compound structures, which consists of two types of unit cells. It was the closest approach to the theoretical maximum ever reported. Gao et al. [62] employed the parametric level set method to design lattice structures, achieving maximum bulk modulus, maximum shear modulus, and a combination of both. Zheng et al. [63] applied the BESO method to design lattice structures with a negative Poisson’s ratio. Song et al. [64] employed the same method to design lattice structures resistant to uniaxial compression loads. Mao et al. [65] developed an inverse homogenization method that extracts the equivalent Hooke’s tensor by minimizing displacement field errors between full-scale simulations and static compression/shear experiments, ultimately identifying an anisotropic constitutive model for homogenized solids. Belardi et al. [66] proposed a calibrated beam FE model based on Kelvin cells. Elastic properties of nodal connections are optimized via the NSGA-II algorithm, establishing an orthotropic homogenized material with high computational efficiency. Zhao et al. [67] proposed a method for mimicking crystal defects to design lattice structures. It can simultaneously improve the mechanical properties and energy absorption of the lattice structure.

The compressive property is one of the important properties of lattice structures. Realizing maximizing bulk modulus design can effectively improve the compressive property. At present, most of the lattice structures exhibit anisotropic property. While TO methods for cubic-symmetric lattices achieve high bulk modulus, their intrinsic anisotropy fundamentally limits applications under multidirectional loading [3,32]. Furthermore, isotropic designs sacrifice stiffness in a previous paper [1]. Therefore, the design of a cubic lattice with isotropy has a certain value. Meanwhile, topology optimization exhibits high efficiency in contrast with the traditional lattice design [45,53]. As a result, this paper proposes the use of topology optimization methods to design lattice structures with isotropy with the aim of maximizing the bulk modulus. The structural material used for the lattice structure is Tough 2000, which exhibits superior mechanical properties under various conditions, including compression, tension, bending, and impact [68].

In this paper, isotropic topology-optimized lattice structures were designed using Abaqus, coupled with homogenization and BESO, to explore optimal configuration. By adjusting the filtering radius, several lattice structures were designed. Subsequently, quasi-static compression tests were conducted on the 3D-printed lattice specimens to determine their mechanical properties. These properties were then compared with those of two traditional lattice configurations. Afterwards, the loading direction dependency of two typical topology-optimized lattice structures was discussed in detail using finite element analysis. Finally, some conclusions were summarized.

## 2. Materials and Methods Topology Optimization Method Based on Abaqus

In this section, the topology optimization for maximizing the bulk modulus of lattice structures is presented, which adopts a homogenization method combining periodic boundary conditions (PBCs) and strain energy-based calculation. To ensure theoretical rigor (consistent with periodic media assumptions) and computational efficiency, PBCs are applied using an Abaqus plugin [69,70,71]. The strain energy density is used to derive the macroscopic effective stiffness matrix and bulk modulus, enabling direct coupling with the BESO algorithm. Aim to maximize the bulk modulus of the lattice structure while considering only volume constraints, as well as volume and elastic isotropy constraints.

**Figure 1:**
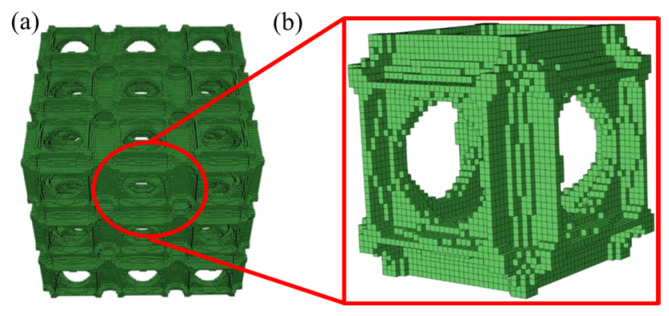
(**a**) Lattice structure; (**b**) RVE.

Generally, the lattice structures are periodic. The smallest repeating structure in two or three dimensions is called a representative volume element (RVE), as shown in Figure 1.

Meanwhile, the lattice structures are usually cubic symmetric. Accordingly, the equivalent macroscopic elastic matrix of RVE can be written as follows [65,66]:

(1) $$C^{H}=\left[\begin{array}{cccccc} C_{11}^{H} & C_{12}^{H} & C_{12}^{H} & 0 & 0 & 0\\ & C_{11}^{H} & C_{12}^{H} & 0 & 0 & 0\\ & & C_{11}^{H} & 0 & 0 & 0\\ & & & C_{44}^{H} & 0 & 0\\ & \mathrm{sym} & & & C_{44}^{H} & 0\\ & & & & & C_{44}^{H} \end{array}\right],$$

where $C_{\mathrm{ij}}^{H}$ represents the homogenization variables. Similar to Equation (1), the average stress vector $\bar{\sigma }$ and the average strain vector $\bar{\varepsilon }$ can be linked together by the equivalent macroscopic elastic matrix

(2) $$\bar{\sigma }=C^{H}\cdot \bar{\varepsilon }$$

According to the above equation, the macroscopic strain energy density of RVE can be expressed as

(3) $$\begin{array}{cc} w & =\frac{1}{2}\bar{\sigma }\cdot \bar{\varepsilon }\\ & =\frac{1}{2}{\bar{\varepsilon }}^{T}\cdot C^{H}\cdot \bar{\varepsilon } \end{array}$$

It is found from the Equation (3) that the equivalent macroscopic elastic matrix of RVE can be calculated by the macroscopic strain energy density. The independent components of CH can be calculated by three independent macroscopic strain states, i.e., ${\bar{\varepsilon }}_{1}={\left[1,\;0,\;0,\;0,\;0,\;0\right]}^{T}$, ${\bar{\varepsilon }}_{2}={\left[1,\;1,\;0,\;0,\;0,\;0\right]}^{T}$, and ${\bar{\varepsilon }}_{3}={\left[0,\;0,\;0,\;1,\;0,\;0\right]}^{T}$. Taking $C_{11}^{H}$ as an example, the calculation of $C^{H}$ is illustrated. Substituting ${\bar{\varepsilon }}_{1}={\left[1,\;0,\;0,\;0,\;0,\;0\right]}^{T}$ into Equation (3), we can obtain that

(4) $$\begin{array}{cc} w^{11} & =\frac{1}{2}{\bar{\varepsilon }}_{1}^{T}\cdot C^{H}\cdot {\bar{\varepsilon }}_{1}\\ & =\frac{1}{2}C_{11}^{H} \end{array}$$

The remaining components can also be calculated from another two independent macroscopic strain states. After obtaining the macroscopic elastic matrix of the lattice RVE, the effective bulk modulus *K*, the effective shear modulus *G*, and the effective elastic modulus *E* can be achieved as

(5) $$\begin{array}{cc} K & =\frac{1}{9}\left(C_{11}^{H}+C_{12}^{H}+C_{13}^{H}+C_{21}^{H}+C_{22}^{H}+C_{23}^{H}+C_{31}^{H}+C_{32}^{H}+C_{33}^{H}\right)\\ & =\frac{1}{9}\left(3C_{11}^{H}+6C_{12}^{H}\right)\\ & =\frac{1}{3}\left(C_{11}^{H}+2C_{12}^{H}\right) \end{array}$$

(6) $$\begin{array}{cc} G & =\frac{1}{3}\left(C_{44}^{H}+C_{55}^{H}+C_{66}^{H}\right)\\ & =\frac{1}{3}\left(3C_{44}^{H}\right)\\ & =C_{44}^{H} \end{array}$$

(7) $$E=\frac{{\left(C_{11}^{H}\right)}^{2}+C_{11}^{H}C_{12}^{H}-2{\left(C_{11}^{H}\right)}^{2}}{C_{11}^{H}+C_{12}^{H}}$$

If the lattice structure is not only cubic symmetric but also elastically isotropic, the three variables $C_{11}^{H}$, $C_{12}^{H}$ and $C_{44}^{H}$ in Equation (1) will no longer be independent in pairs. The macroscopic equivalent elastic matrix of the isotropic lattice structure RVE can be written as

(8) $$\left[\begin{array}{cccccc} C_{11}^{H} & C_{12}^{H} & C_{12}^{H} & 0 & 0 & 0\\ & C_{11}^{H} & C_{12}^{H} & 0 & 0 & 0\\ & & C_{11}^{H} & 0 & 0 & 0\\ & & & \frac{C_{11}^{H}-C_{12}^{H}}{2} & 0 & 0\\ & sym & & & \frac{C_{11}^{H}-C_{12}^{H}}{2} & 0\\ & & & & & \frac{C_{11}^{H}-C_{12}^{H}}{2} \end{array}\right]$$

Comparing Equations (1) and (8), it can be found that the elastic component $C_{44}^{H}$ of isotropic lattice structure can be expressed by $C_{11}^{H}$ and $C_{12}^{H}$ as $C_{44}^{H}\;=\;(C_{11}^{H}\;-\;C_{12}^{H})/2$. Without loss of generality, the relationship between the three components is rewritten as

(9) $$\begin{array}{l} 2\left(C_{11}^{H}+C_{22}^{H}+C_{33}^{H}\right)-\left(C_{12}^{H}+C_{13}^{H}+C_{21}^{H}+C_{23}^{H}+C_{31}^{H}+C_{32}^{H}\right)\\ -4\left(C_{44}^{H}+C_{55}^{H}+C_{66}^{H}\right)=0 \end{array}$$

where $C_{22}^{H}\;={\;C}_{33}^{H}\;=\;C_{11}^{H}$, $C_{13}^{H}\;=\;C_{21}^{H}=\;C_{23}^{H}\;=\;C_{31}^{H}\;=\;C_{32\;}^{H}=\;C_{12}^{H}$, $C_{55}^{H}\;=\;C_{66}^{H}\;=\;C_{44}^{H}$.

Zener anisotropy index was proposed by Zener [72,73,74] to quantify the anisotropy properties of cubic crystals. The Zener anisotropy index is defined as

(10) $$A=\frac{2C_{44}^{H}}{C_{11}^{H}-C_{12}^{H}}$$

where *A* represents the anisotropy index.

### 2.1. Optimization of Maximizing Bulk Modulus Considering Volume Constraints

The calculation procedure based on BESO is established for the optimization problem of maximizing bulk modulus under two different types of constraints, i.e., the volume constraint and the combined volume and isotropic constraint. The optimization problem of maximizing bulk modulus considering volume constraints can be described as follows:

(11) $$\begin{array}{l} Maximize\;\;\;f=K\\ Subject\;\;\;to:V^{*}-\sum_{i=1}^{n}V_{i}x_{i}=0\\ \;\;\;\;\;\;\;\;\;\;\;\;\;\;\;\;\;\;\;\;\;\;\;\;\;\;\;x_{i}=x_{\min}\;\;or\;\;1 \end{array}$$

where f represents the objective function to be optimization, $V^{*}$ represents the target volume, $V_{i}$ represents the volume of a single element, and $x_{i}$ represents the element density which can be 1 or $x_{min}$. Here, $x_{min}$ is taken as 0.001.

To enable finite element analysis, solid isotropic material with penalization (SIMP) is employed to define the effective elastic modulus assigned to each element. The elastic modulus of the element after interpolation is

(12) $$E\left(x_{i}\right)=E_{1}x_{i}^{p}$$

where $E_{1}$ represents the Young’s modulus of the matrix material which is taken as 1 in this paper, and p represents the penalty index which is taken as 3.

It can be found that bulk modulus *K* and shear modulus *G* [75] can be expressed by the components of the macroscopic equivalent elastic matrix. The components of the macroscopic equivalent elastic matrix can be expressed by the strain energy density. Accordingly, the equivalent bulk modulus *K* and equivalent shear modulus *G* can also be fully expressed by the strain energy density. The sensitivity number of the strain energy *C* of the lattice representative volume element (RVE) to the element *i* can be expressed as

(13) $$\frac{dC}{dx_{i}}=\frac{1}{2}u_{i}^{T}px_{i}^{p-1}k_{0}u_{i}=\frac{p}{x_{i}}C_{i}$$

where $u_{i}$ is the element displacement vector, $k_{0}$ is the element stiffness matrix for an element with an element density of 1, and $C_{i}$ is the strain energy of the element. From the above formula, the sensitivity number of the strain energy density of RVE to the element *i* is

(14) $$\frac{dw}{dx_{i}}=\frac{1}{V}\frac{p}{x_{i}}C_{i}=\frac{v_{i}}{V}\frac{p}{x_{i}}w_{i}$$

where *w* is the macroscopic strain energy density, *V* is the volume of RVE, $v_{i}$ is the volume of element *i*, and $w_{i}$ is the strain energy density of element *i*. According to Equation (14), the sensitivity of the optimization objective function to the element *i* can be calculated as

(15) $$\alpha_{i}=\frac{dK}{dx_{i}}$$

where *K* is the bulk modulus.

To avoid the checkerboard effect in the optimization results and reduce the grid dependence, a filtering algorithm [76] is used to correct the initial sensitive number of the element (Equation (15)), and the filtered sensitive number is expressed as

(16) $${\bar{\alpha }}_{i}=\frac{\sum_{j}w\left(d_{ij}\right)\alpha_{j}}{\sum_{j}w\left(d_{ij}\right)}=\sum_{j}\xi_{j}\alpha_{j}$$

Among them, $\xi_{j}$ represents the influence weight of the sensitive number of element *j* to the sensitive number of element *i*. $w(d_{\mathrm{ij}})$ is the weight function of element *j* to element *i* within the filter radius $r_{filter}$, which can be denoted as

(17) $$w\left(d_{ij}\right)=\max\left(0,r_{filter}-d_{ij}\right)$$

where $d_{\mathrm{ij}}$ is the distance between two element centers.

To make the optimization process more stably and improve the convergence of the algorithm, the historical average algorithm for sensitive numbers proposed by Huang and Xie [77] is used,

(18) $${\hat{\alpha }}_{i}=\frac{{\bar{\alpha }}_{i}^{k}+{\bar{\alpha }}_{i}^{k-1}}{2}$$

Each optimization starts from the full design domain, and the volume after each iteration is

(19) $$V^{k+1}=V^{k}\left(1-ER\right)$$

Here, $V^{k}$ represents the volume of the current iteration step, $V^{k+1}$ is the volume of the next iteration step, and *ER* represents the evolutionary ratio. The final expression for the volume of the next iteration step is

(20) $${\hat{V}}^{k+1}=\max\left(V^{k+1},V^{*}\right)$$

The sensitivity numbers according to Equation (18) are done, and then the sensitivity numbers are sorted in descending order. The final volume of the next iteration according to Equation (20) is calculated in order to determine the threshold value of whether the cell should be a solid cell or an empty cell in the next iteration step. Accordingly, the topology configuration of the lattice RVE can be updated.

**Figure 2:**
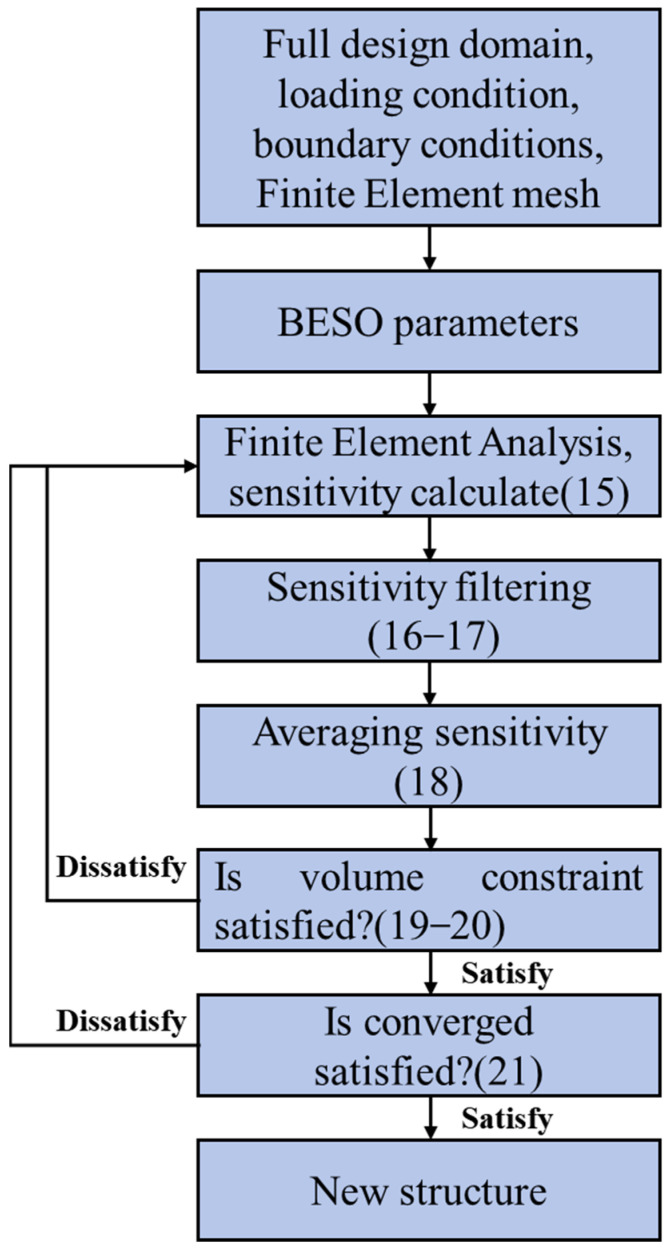
Optimization flowchart of maximizing bulk modulus considering volume constraints.

When the target volume is satisfied in the iteration, the termination condition of the whole calculation loop is determined by the following convergence criteria

(21) $$error=\frac{\left|\sum_{i=1}^{5}f_{k-i+1}-\sum_{i=1}^{5}f_{k-i-4}\right|}{\sum_{i=1}^{5}f_{k-i+1}}\leq \tau$$

where *τ* is the acceptable convergence error (convergence tolerance), which is taken as 0.001 here. The optimization flowchart is shown in Figure 2.

### 2.2. Optimization of Maximizing Bulk Modulus Considering Volume and Isotropic Constraints

The mathematical formulation of the optimization problem regarding maximizing bulk modulus under both bulk and isotropic constraints is given as

(22) $$\begin{array}{l} Maximize\;\;\;f=K\\ Subject\;\;\;to:V^{*}-\sum_{i=1}^{n}V_{i}x_{i}=0\\ \;\;\;\;\;\;\;\;\;\;\;\;\;\;\;\;\;\;\;\;\;\;\;\;\;\;\;C_{iso}=0\\ \;\;\;\;\;\;\;\;\;\;\;\;\;\;\;\;\;\;\;\;\;\;\;\;\;\;\;x_{i}=x_{\min}\;\;\;or\;\;\;1 \end{array}$$

where $C_{\mathrm{iso}}$ is the isotropic constraint, and its specific form is Equation (9).

Huang and Xie [77] indicated that in the BESO optimization algorithm, other constraints except for the volume constraint can be introduced into the objective function by Lagrange multipliers. After using this method, the isotropic constraint is introduced into the objective optimization function, and the equivalent objective function becomes

(23) $$f_{1}=f+\Lambda C_{iso}$$

where Λ∈(−∞, +∞) represents the Lagrange multiplier. To determine the value range of Λ conveniently, rewriting the equivalent form of Equation (23) as [78]

(24) $$f_{1}=\left(1-\left|\lambda \right|\right)f+\lambda C_{iso}$$

where λ∈(−1, 1). Consequently, the optimization problem described by Equation (22) can be written in the following form

(25) $$\begin{array}{l} Maximize\;\;\;f_{1}=\left(1-\left|\lambda \right|\right)K+\lambda C_{iso}\\ Subject\;\;\;to:V^{*}-\sum_{i=1}^{n}V_{i}x_{i}=0\\ \;\;\;\;\;\;\;\;\;\;\;\;\;\;\;\;\;\;\;\;\;\;\;\;\;\;\;x_{i}=x_{\min}\;\;\;or\;\;\;1 \end{array}$$

The sensitivity of the objective function $f_{1}$ to the element density $x_{i}$ is

(26) $$\alpha_{i}=\frac{df_{1}}{dx_{i}}=\left(1-\left|\lambda \right|\right)\frac{dK}{dx_{i}}+\lambda \frac{dC_{iso}}{dx_{i}}$$

**Figure 3:**
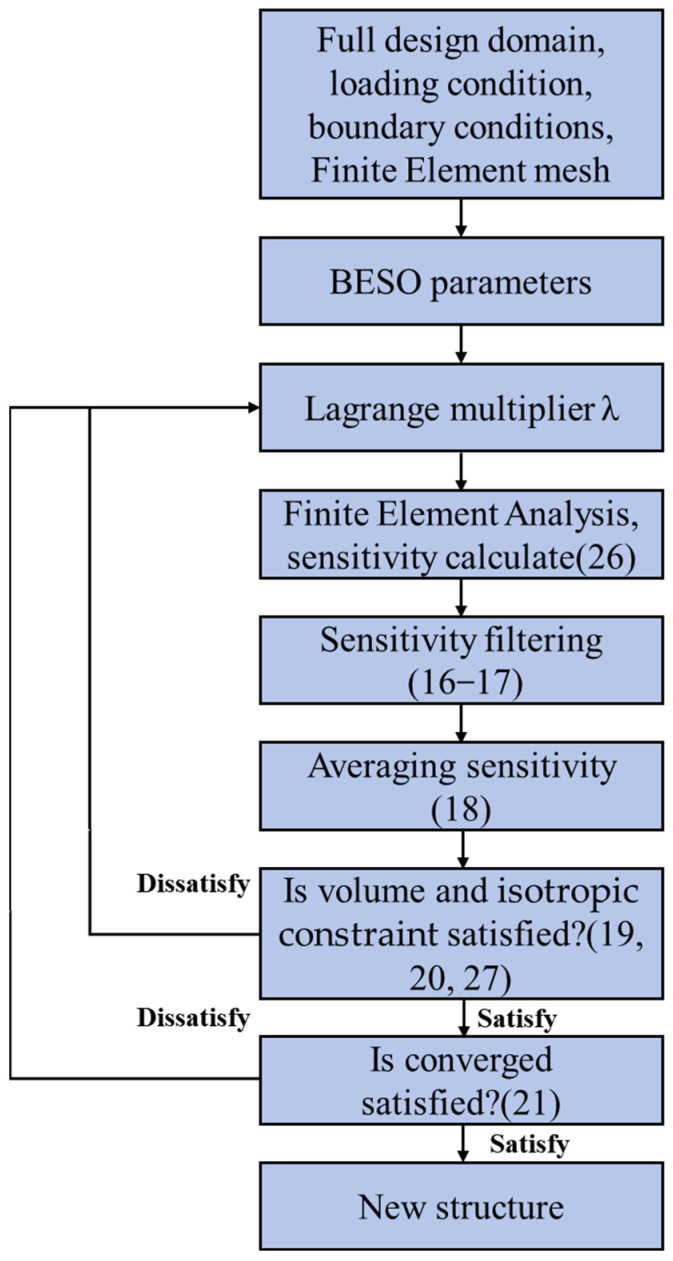
Optimization flowchart of maximizing bulk modulus considering volume and isotropic constraints.

Since both *K* and $C_{\mathrm{iso}}$ can be expressed by the strain energy density, the sensitivity of the equivalent objective function $f_{1}$ to the element density can be solved by Equation (14). Equation (26) contains the Lagrange multiplier λ, so the value of the Lagrange multiplier needs to be determined before calculating the sensitive number of the element. The Lagrange multiplier can be determined as the isotropic condition $C_{\mathrm{iso}}^{k+1}$ at the next iteration step is closer to 0 than the isotropic condition $C_{\mathrm{iso}}^{k}$ at the current iteration step [78], which means $\left|C_{\mathrm{iso}}^{k+1}\right|\;\leq \;\left|C_{\mathrm{iso}}^{k}\right|$. The value of the isotropic constraint of the next iteration step can be approximated by the value of the isotropic constraint of the current iteration step combined with the next topology configuration, and its expression is

(27) $$C_{iso}^{k+1}\approx C_{iso}^{k}+\sum_{i=1}^{n}\frac{dC_{iso}^{k}}{dx_{i}}\Delta x_{i}$$

where $C_{\mathrm{iso}}^{k+1}$ is the estimated value of the isotropic constraint for the next configuration, $C_{\mathrm{iso}}^{k}$ is the value of the isotropic constraint corresponding to the current configuration, and $\Delta x_{i}$ is the variation in element density of element *i* between the configuration at the current iteration step and that at the next iteration step. When determining the Lagrange multiplier, the λ is initially set to 0, and then it is substituted into Equation (26) to obtain the sensitivity number of the equivalent optimization objective function to the element. Finally, Equations (16) and (18) are employed to process the initial element sensitivity numbers. The topology configuration of the lattice RVE is updated according to the volume constraints Equation (20). Afterwards, $C_{\mathrm{iso}}^{k+1}$ is calculated by Equation (27). If $C_{\mathrm{iso}}^{k+1}\;\geq \;0$, the final λ is determined between (−1, 0]. Otherwise, if $C_{\mathrm{iso}}^{k+1}\;\leq \;0$, the final λ is between [0, 1). Repeating the above-mentioned value-determination process continuously until the length of the region is not larger than 1 × 10^−5^, then the value-determination process is terminated and the Lagrange multiplier λ can be determined. When the convergence criterion Equation (21) is satisfied, the entire optimization process is finished, and the final topology configuration of the lattice structure RVE can be obtained. The optimization flowchart is shown in Figure 3.

## 3. Design and Experiments

The process for two types of optimization problems was established previously. In this section, these two types of optimization problems will be solved by Python (3.10.4) code. And the corresponding lattice configurations will be generated by the optimization process in Section 2. Afterwards, compression tests are performed to determine the mechanical properties of the topology-optimized lattice structures.

### 3.1. Structural Design of Topology Optimization Lattice Structure

**Figure 4:**
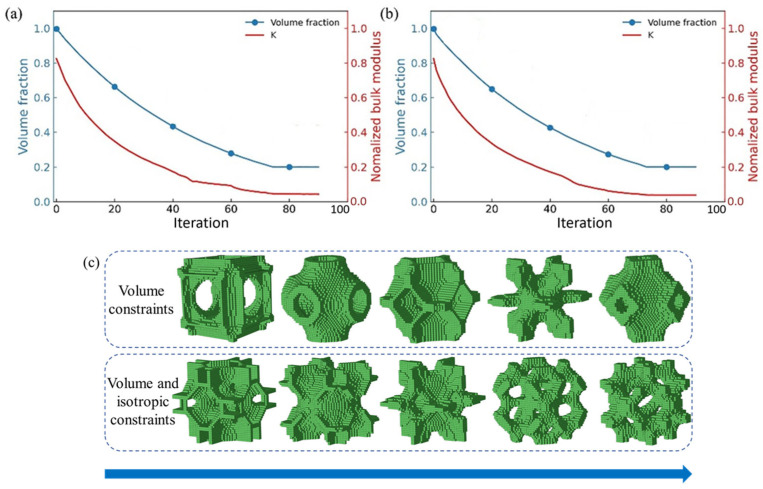
Evolution history of bulk modulus and volume fraction during optimization: (**a**) considering only volume constraints, (**b**) considering both volume and isotropic constraints, (**c**) evolution of lattice structure configuration.

To quantify the anisotropic properties of cubic crystals, Zener proposed the anisotropy index [72], which has been widely used in many studies [72,73,79]. Figure 4 shows the evolution history of the bulk modulus and volume fraction of the lattice structure when the iterations increase from 0 to 80. The evolution ratio *ER* in the figure is 0.02, while the $r_{\mathrm{filter}}$ is 2.6 and the target volume fraction $V_{f}$ is 0.2. Here, Figure 4a only considers the volume constraint, while Figure 4b considers both volume and isotropic constraints. Figure 4a,b presents that the bulk modulus and volume fraction of the lattice structure gradually decrease with the iterations. When the volume fraction constraint is satisfied, the entire optimization process is completed. It can be observed that the solid part of the configuration gradually decreases with the increase in iterations (follow the blue arrow indication), as exhibited in Figure 4c.

**Figure 5:**
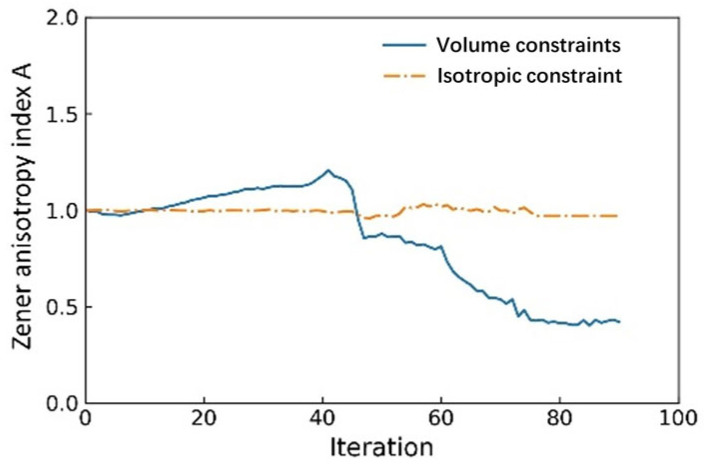
The evolution of anisotropy index during the iterations.

Figure 5 shows the evolution of the anisotropy index of RVE with the iterations. For the isotropic constraint (the orange dash-dot line), the anisotropy index fluctuates slightly around 1. For the volume constraints (the blue line), the fluctuation range of the anisotropy index is larger, ranging from above 1 to below 1. It can be observed that the anisotropic properties of lattice structures can be effectively controlled by introducing elastically isotropic constraints. Therefore, the elastically isotropic lattice structure can be obtained through the topology optimization method.

**Figure 6:**
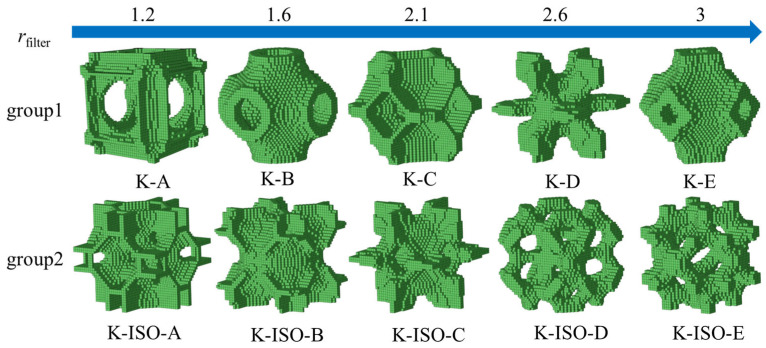
The microscopic structure of topology optimization lattice structures under different filtering radii.

The filter radius was proposed in the classical BESO optimization method to reduce the dependence of the optimization results on the mesh and avoid the checkerboard effect [80]. Duan et al. [72] used the filter radius as one of the optimization parameters, resulting in diverse lattice structures. In this work, maximum bulk modulus is selected as the objective function. Different filter radii, including 1.2, 1.6, 2.1, 2.6, and 3, are selected for optimization to obtain different lattice structures, as shown in Figure 6. The target volume fraction is 0.2, the evolution ratio *ER* = 0.02, and the penalty index is *p* = 3. Group 1 represents the topology-optimized lattice structure with isotropic constraint, while Group 2 represents the topology-optimized lattice structure with volume constraints. It is apparent that all these lattice RVEs exhibit cubic symmetry, which is consistent with Huang [75]. Additionally, it can also be found that the configurations of these RVEs contain abundant basic constituent characteristics. For example, the structures of K-A, K-C, and K-D are mainly composed of plates, while K-B and K-E are mainly composed of shells. In group 2, K-ISO-A, K-ISO-B, and K-ISO-C are mainly composed of plates and shells, while K-ISO-D and K-ISO-E are mainly composed of rods. The topology optimization results can obtain structures similar to those of the previous studies. For example, K-B is similar to the triply periodic minimal surface Schwarz P structure [81]. Moreover, the topology optimization results can also obtain novel lattice structures that have not been studied in previous studies.

### 3.2. Preparation of Topology-Optimized Lattice Structure

In this section, RVEs of 10 different lattice structures are obtained by the topology optimization method. The structure has a step-like coarse surface, which is because BESO uses the element density $x_{i}$ to describe the presence or absence of elements.

**Figure 7:**
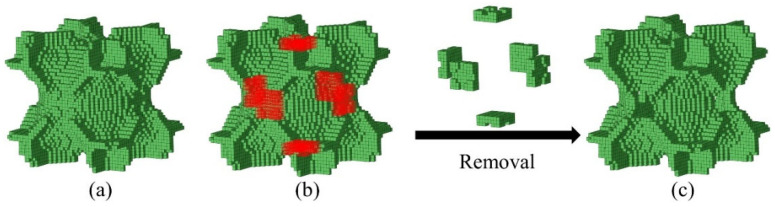
Modification for K-ISO-B configuration: (**a**) original K-ISO-B configuration, (**b**) removed parts (marked in red), (**c**) final configuration.

The macroscopic elastic matrix of the RVE is calculated from the strain energy density of the element. Changing the geometry of the RVE may affect its macroscopic mechanical properties. Accordingly, no additional processing is performed on the original geometry when preparing the specimen. It should be noted that the K-ISO-B configuration forms a closed geometric space; the closed geometric spaces can trap resin or air during the fabrication process. Therefore, it does not meet the SLA fabrication requirements. To ensure that K-ISO-B can be successfully fabricated, the structure is modified as shown in Figure 7. Figure 7a is the original K-ISO-B configuration, while the red elements marked in Figure 7b need to be removed. Figure 7c is the final configuration obtained after removing the marked elements, which is noted as K-ISO-B-m and used for subsequent fabrication. Except for minor adjustments to K-ISO-B, other topology-optimized geometries are directly manufactured without post-processing.

**Figure 8:**
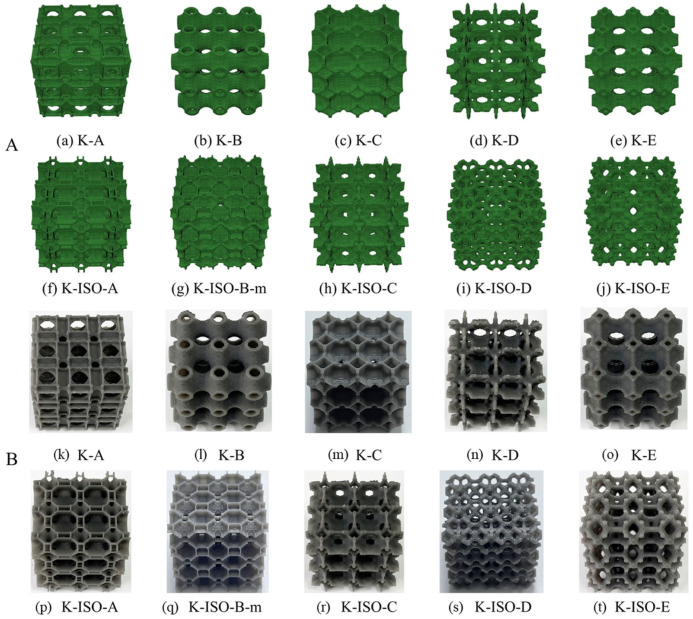
(**A**) Different geometric model of the topology-optimized lattice structures for (**a**–**j**), (**B**) samples corresponding to different lattice structures fabricated by SLA for (**k**–**t**).

The RVEs are extended along three spatially principal directions in Abaqus to obtain complete lattice structures. These lattice structures are manufactured by the SLA method on a Form 3 printer. In this work, the matrix material is Tough 2000 resin (Formlabs, Somerville, MA, USA). The Tough 2000 material exhibits an elastic modulus *E* = 1897.63 MPa, a Poisson’s ratio of 0.3, and a density of 1100 kg/m^3^. The Form 3 printer is equipped with a light processing unit. The laser spot size is 85 μm, and the laser power is 250 mW. The resolution in the x-y plane is 25 μm, and the adopted layer thickness is 50 μm. Figure 8A illustrates the geometries of all the lattice structures employed for fabrication, while Figure 8B displays the fabricated lattice samples. Attributed to the high printing accuracy, no obvious geometric defects are observed in the samples. Consequently, the influence of geometric defects on the mechanical properties of the printed lattice specimens can be ignored in the finite element models.

**Figure 9:**
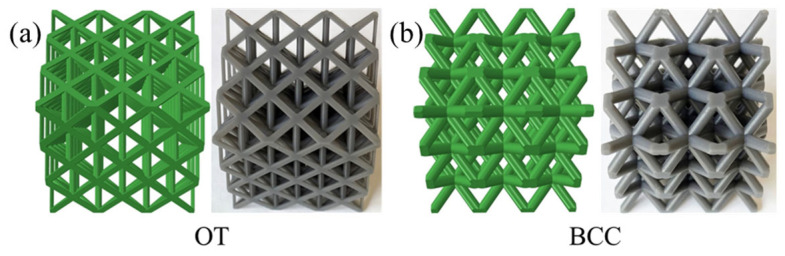
The geometric models (green type) and as-fabricated specimens (grey type) of the traditional typical lattice structure: (**a**) OT lattice structure, (**b**) BCC lattice structure.

Moreover, two types of representative lattice structures, including OT and BCC, are selected for comparison. The two traditional lattice structures have different dominant deformation behaviors. The OT is dominated by stretching in struts, which is suitable for load bearing, as shown in Figure 9a. While the BCC is dominated by strut bending, which is usually used for energy absorption, as shown in Figure 9b. Figure 9 presents the geometric models of the two conventional lattice structures as well as the additively manufactured samples. Compared with the specimens in Figure 8B, the specimens in Figure 9 exhibit a smoother surface. This is because the geometric models of the OT and the BCC lattice structures are directly built by computer-aided design software (Solidworks 2018) with smooth surfaces.

An electronic balance with an accuracy of 0.01 g is used to measure the mass of the additively manufactured lattice structure. A digital caliper with an accuracy of 0.01 mm is adopted to measure the three-dimensional geometric dimensions of the lattice structure. Two samples for each type of the lattice structure are prepared for repeated experiments. The measured results are shown in Table 1.

### 3.3. Compression Tests on the Topology-Optimized Lattice Structures

**Figure 10:**
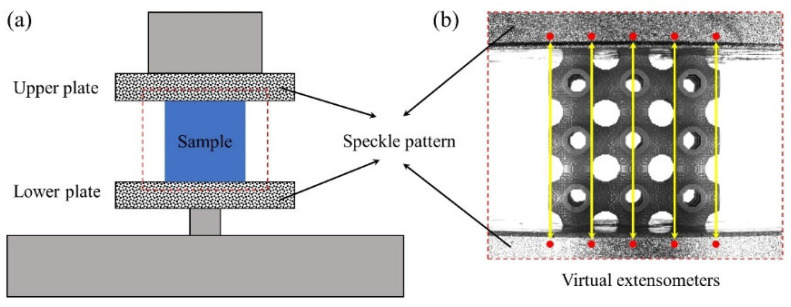
Schematic diagram of measuring the compression displacement: (**a**) schematic diagram of quasi-static compression device; (**b**) virtual extensometer.

The additively manufactured lattice structures are tested on an electronic universal testing machine (CMT4104) (Shenzhen SANS Testing Machine, Shenzhen, China) to obtain their quasi-static mechanical properties. The range of the force sensor on the electronic universal testing machine is 10 kN. The nominal strain rate of the tests is 0.001 s^−1^. Accordingly, the corresponding loading rate is 0.036 mm⋅s^−1^. An industrial camera (MER-503-36U3M) (Daheng Imaging, Beijing, China) is placed in front of the specimens to capture the deformation evolution process of the lattice structures. The acquisition frequency of the camera is 1 fps. In order to obtain the accurate compression deformation of the lattice structure in the elastic stage, speckles are sprayed on the upper and lower indenters of CMT4104. Subsequently, the actual compression distance of the indenter can be obtained using the digital image correlation (DIC) technology. Figure 10 exhibits the schematic diagram of displacement measurement during the quasi-static compression tests.

### 3.4. Finite Element Analysis

**Figure 11:**
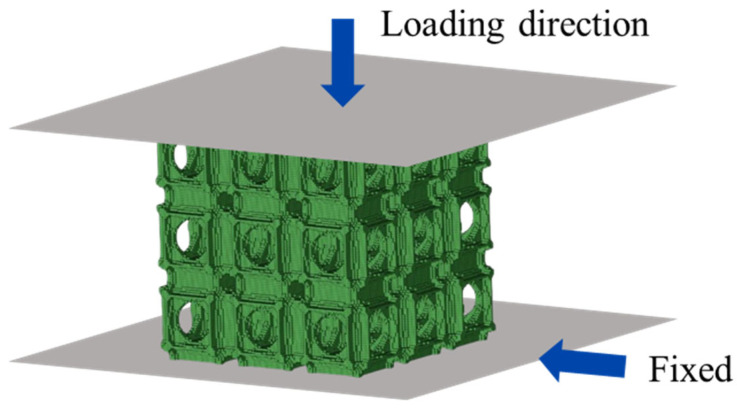
Schematic of finite element model lattice.

Finite element analysis is necessary for reliably predicting the mechanical behavior of lattice structures under a quasi-static loading state. This method can observe the complex deformation mechanisms of topology-optimized lattice structures. In addition to experiments, numerical simulations were also supplemented to evaluate the loading direction dependence of the topology-optimized lattice structures. Abaqus software (6.14-4) has strong advantages in handling numerical simulations of large nonlinear deformations. Therefore, the Abaqus/Explicit solver is used for solving [82]. A series of finite element models were established using the commercial software Abaqus, as shown in Figure 11. The lattice structures were discretized using 3D solid elements (C3D8R in Abaqus). The loading process was controlled by two rigid plates, where the upper plate moved downwards with a nominal strain rate of 0.001 s^−1^ and all degrees of freedom of the bottom panel were constrained. General contact with a friction coefficient of 0.3 was adopted between the lattice structure and rigid plates as well as the inner struts of the lattice. The Von-Mises yield function accompanied by the isotropic hardening model was adopted to characterize the basis material in the simulations. The effective stress versus plastic strain data of the basis material obtained from experiments was directly input into the software, which have been denoted in our previous work comprehensively [78].

## 4. Results and Discussion

In this section, the results regarding the mechanical properties and failure modes of the additively manufactured lattices are presented through quasi-static compression tests and finite element simulations. Furthermore, the topology-optimized lattices are compared with the traditional lattices, and some intrinsic mechanisms are discussed.

### 4.1. Elastic Properties of the Topology-Optimized RVEs

**Figure 12:**
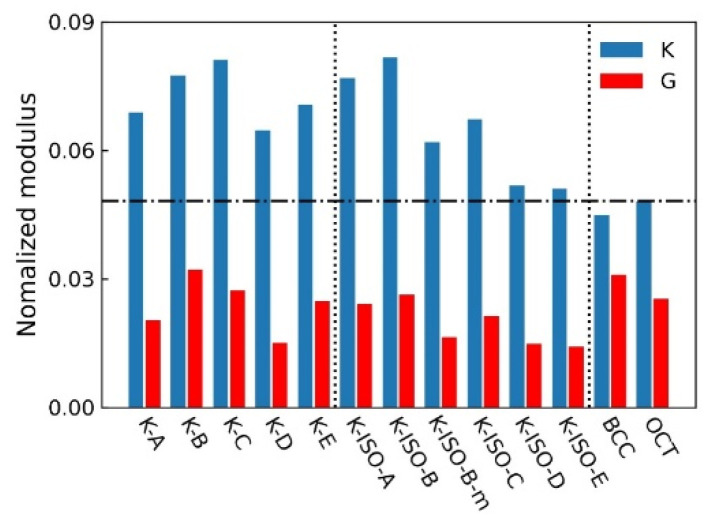
Normalized bulk and shear moduli of RVEs (The dotted lines represent different types of lattice structures and dashed line represents the *K* of OCT.).

The bulk modulus and shear modulus of the topology-optimized lattice RVEs can be obtained during the optimization process. Figure 12 shows the bulk and shear modulus of the RVEs normalized using *E* = 1. The blue and red bars represent the normalized bulk modulus and shear modulus of the lattice structures. It can be observed that all bulk modulus *K* obtained by topology optimization are higher than the moduli of BCC and OT. Especially, it is worth noting that when the bulk modulus is taken as the optimization objective, the bulk modulus *K* of the lattice structure is higher than its shear modulus *G*. As a result, the mechanical properties of the lattice structures designed by the topology optimization method are better than those of the traditional lattice structures, which demonstrates their potential applications.

**Figure 13:**
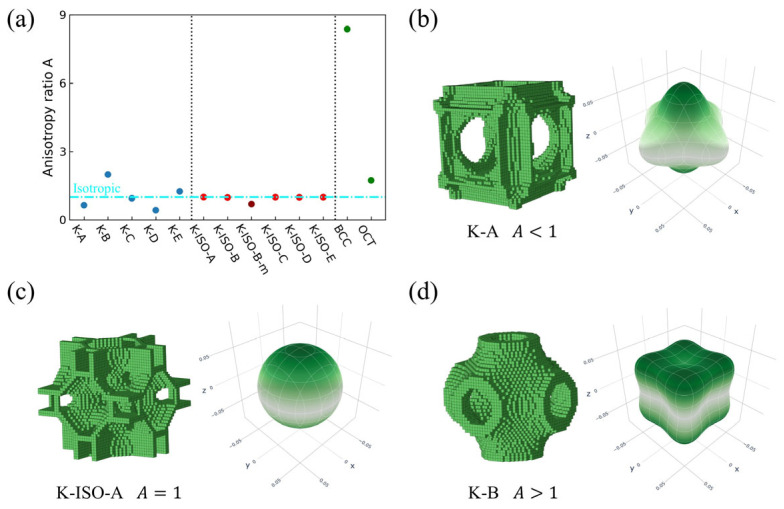
The anisotropy indexes of the lattice structures and the spatial distribution of elastic moduli of three typical topology-optimized lattice structures: (**a**) the anisotropy indexes of the different lattice RVEs (The dotted lines represent different types of lattice structures; dashed line represents A = 1; different colors of dots represent different types of lattice structures.); (**b**) spatial distribution of elastic modulus for K-A lattice (the anisotropy index is less than 1); (**c**) spatial distribution of elastic modulus for K-ISO-A lattice (the anisotropy index is equal to 1); (**d**) spatial distribution of elastic modulus for K-B lattice (the anisotropy index greater than 1).

Figure 13 shows the anisotropy index of the topology-optimized lattice structures as well as the two traditional lattice structures. Meanwhile, the spatial distribution of elastic modulus for three representative topology-optimized lattice structures is also provided. For the RVEs obtained without considering isotropic constraints, the anisotropy indexes may be greater or less than 1, as presented in Figure 13a. The anisotropy indexes of the RVEs obtained by considering isotropic constraints all fluctuate slightly around 1, while those of the two traditional lattice structures are higher than 1. Under the same relative density, the anisotropy index of BCC is significantly higher than that of the OT. Additionally, the anisotropy index of K-ISO-B-m (Figure 7) is also included in Figure 13a. It can be found that the slight change in the topology makes the anisotropy index decrease from 0.98 (K-ISO-B) to 0.70 (K-ISO-B-m).

From the configurations of the topology-optimized lattice structure, K-A (A < 1), K-ISO-A (A = 1), and K-B (A > 1) are selected, respectively. The spatial distributions of the configurations and their corresponding elastic moduli are plotted in Figure 13b–d [83]. It can be found that when the anisotropy index is lower than 1, the values of the elastic moduli in the three coordinate directions (x, y, and z axes) in space are higher than those in other directions. When the anisotropy index is higher than 1, the values of the elastic moduli in the three spatial coordinate directions (x, y, and z axes) are lower than those in other directions. When the anisotropy index is 1, the elastic modulus is independent of the spatial direction.

### 4.2. Quasi Static Experimental Results of Additively Manufactured Lattice Structures

**Figure 14:**
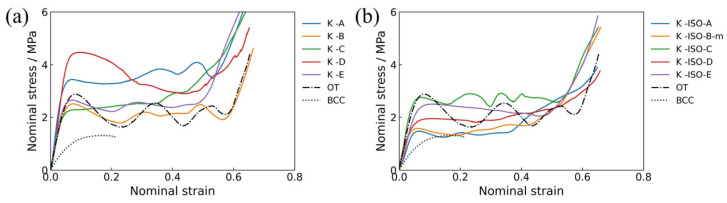
Nominal stress–strain curve of lattice structure under quasi-static loading: (**a**) lattice structure considering volume constraints, (**b**) lattice structure considering both volume and isotropic constraints.

In this section, the deformation and failure modes of topology-optimized lattice structures are analyzed in detail. Figure 14 shows the nominal stress–strain curves for all topology-optimized lattice structures under quasi-static loading. The stress–strain curves of all samples exhibit typical three-stage characteristics, firstly experiencing a transient elastic stage, followed by a long fluctuating stress plateau stage, and finally densification. Figure 14a depicts the nominal stress–strain curve of the lattice structures when only volume constraints are considered. It can be concluded that although the relative densities of the five lattice structures with cubic symmetry are the same, the stress–strain curves present obvious configuration dependence. For simplicity, several representative configurations are selected for discussion. Herein, the K-A lattice structure possesses both high initial peak stress and a stable plateau segment, while the K-C lattice structure exhibits the smallest stress fluctuations in the plateau segment, and the K-D lattice structure has the highest initial peak stress. Comparing the topology-optimized lattice structures with BCC and OT, the elastic moduli of the five topology-optimized lattice structures are much higher than those of BCC, while K-A and K-D are higher than OT. In addition, the elastic modulus and initial peak stress of K-A and K-D are higher than those of OT, which indicates that they are more suitable for lightweight load-bearing fields. Meanwhile, the stress amplitudes of these two lattice structures in the stress plateau stage are also higher than that of OT, which demonstrates that they are also expected to be applied in the field of energy absorption. It is worth noting that the maximum strain of the nominal stress–strain curve for BCC is only around 0.2, which is different from the long and flat stress–strain curves of BCC [84,85]. This phenomenon is due to the obvious collapse of BCC during the compression process, and the local deformation in the lattice structure makes it fracture and completely lose its load-bearing capacity.

Figure 14b displays the nominal stress–strain curves of the lattice structures, which consider both volume and isotropic constraints. For the five topology-optimized lattice structures, the elastic moduli are significantly higher than that of BCC, and K-ISO-C is higher than that of OT. Moreover, the stress plateau stage of the five topology-optimized lattice structures is more stable, and the stress fluctuation amplitude is smaller. Among them, K-ISO-C performs higher in elastic modulus, initial peak stress, and plateau stress.

**Figure 15:**
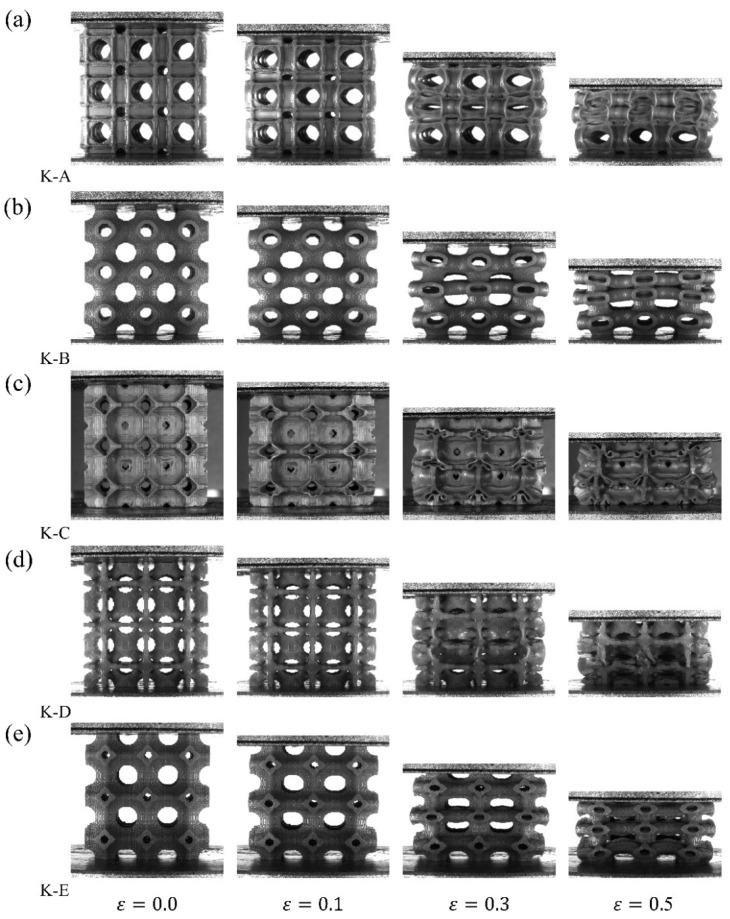
Deformation modes under [1, 0, 0] loading for volume considered topology optimized lattice structure.

**Figure 16:**
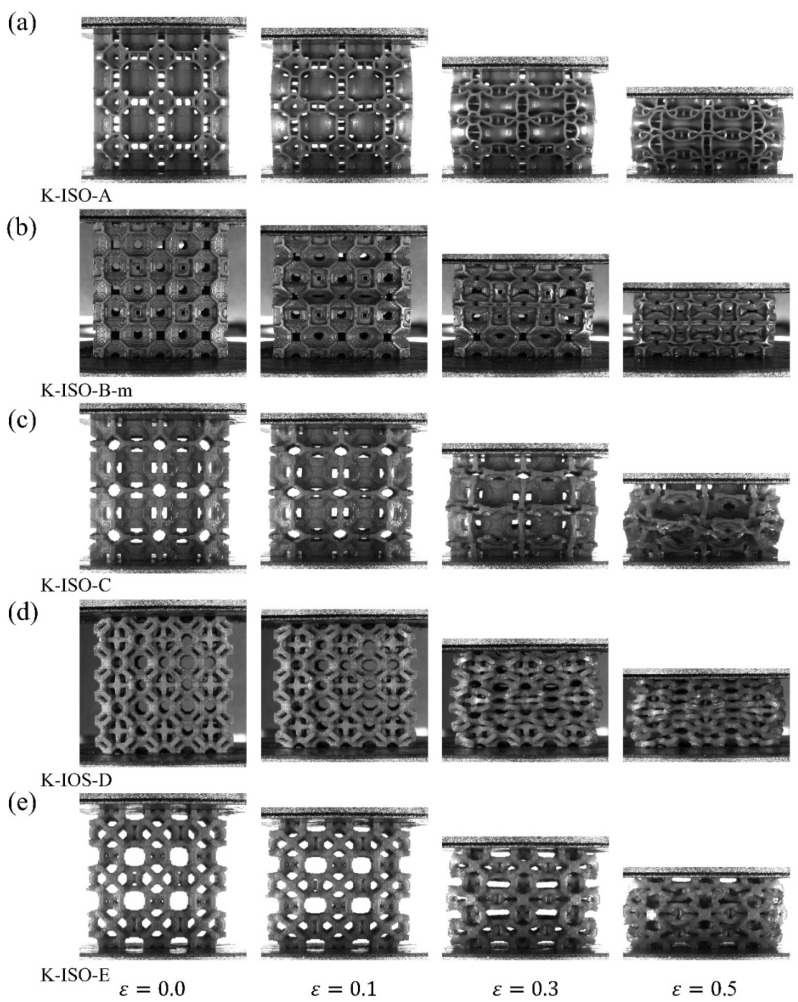
Deformation modes under [1, 0, 0] loading for volume and isotropic considered topology optimized lattice structure.

**Figure 17:**
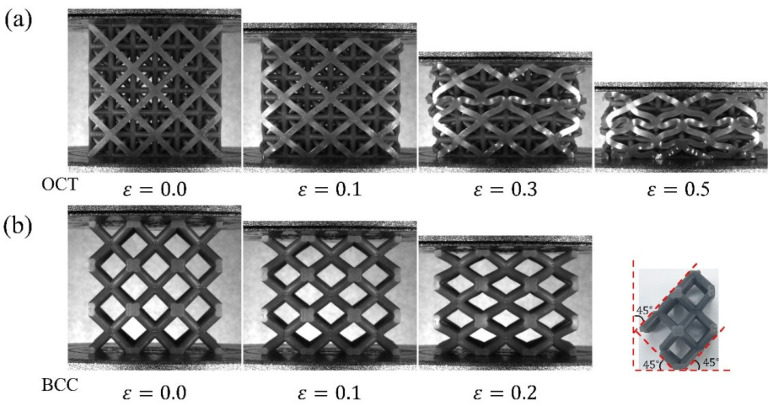
Deformation modes of two traditional lattice structures under [1, 0, 0] loading: (**a**) OT lattice structure; (**b**) BCC lattice structure.

All deformation mode figures (Figure 15, Figure 16 and Figure 17) display results from the [1, 0, 0] directional compression experiment. The deformation and failure modes of the lattice structures obtained by the topology optimization method are presented in Figure 15 when only volume constraints are considered. When the nominal strain is within 0.1, the deformation of the five lattice structures is very uniform. After the nominal strain reaches 0.3, more significant deformation is detected in the middle part of the five lattice structures than in the parts near the upper and lower indenters. This is because the constraint of the middle part in the lattice structure is significantly lower than that at the two ends. Additionally, a slight fracture has appeared in the lattice specimens. When the nominal strain is 0.5, the cell walls of the lattice structure experience extensive contact and fracture, which leads to the densification of the structures.

The deformation modes of the topology-optimized lattice structures under quasi-static compression considering both volume and isotropic constraints are exhibited in Figure 16. Actually, the deformation modes of these lattice structures are similar to those of the lattice structures in Figure 15. When the strain is small, the lattice structures undergo homogeneous deformation from a macroscopic perspective. Afterwards, the middle parts of the lattice structures experience obvious lateral deformation. Ultimately, the cell walls or struts within the lattice structure experience widespread contact.

Figure 17 shows the deformation modes of the two traditional lattice structures, OT and BCC. When the strain is 0.1, some strut members of OT have been buckled as shown in Figure 17a. This corresponds to the elastic stage on the nominal stress–strain curve of the lattice structure. Once the load exceeds the critical load of the lattice structure, the nominal stress–strain curve of the lattice structure begins to decline. When the strain is 0.3, some strut members of OT are in contact, and the concentrated deformation area makes the lattice structure appear as a horizontal deformation band. Finally, all cells of OT are deformed when the strain is 0.5, which corresponds to the last peak on the stress–strain curve of OT. In Figure 17b, no obvious deformation band occurs in BCC before a nominal strain of 0.2. However, when the nominal strain is about 0.22, the BCC lattice structure completely loses its load-bearing capacity due to the severe fracture of the specimen. The larger remnants of the lattice material after failure are shown in Figure 17b. As displayed in the fourth subgraph of Figure 17b, the BCC lattice structure is mainly broken at the nodes. There is no obvious residual deformation on the struts between the two nodes, and the connecting line in the damaged area is along 45°. The failure mode is consistent with the BCC structure in previous studies [84,85,86]. The reason for this deformation mode is that BCC is a typical bending-dominant lattice structure, which means the stress at the nodes is significantly higher than that in the struts.

### 4.3. Mechanical Properties of Topology-Optimized Lattice Structures

**Figure 18:**
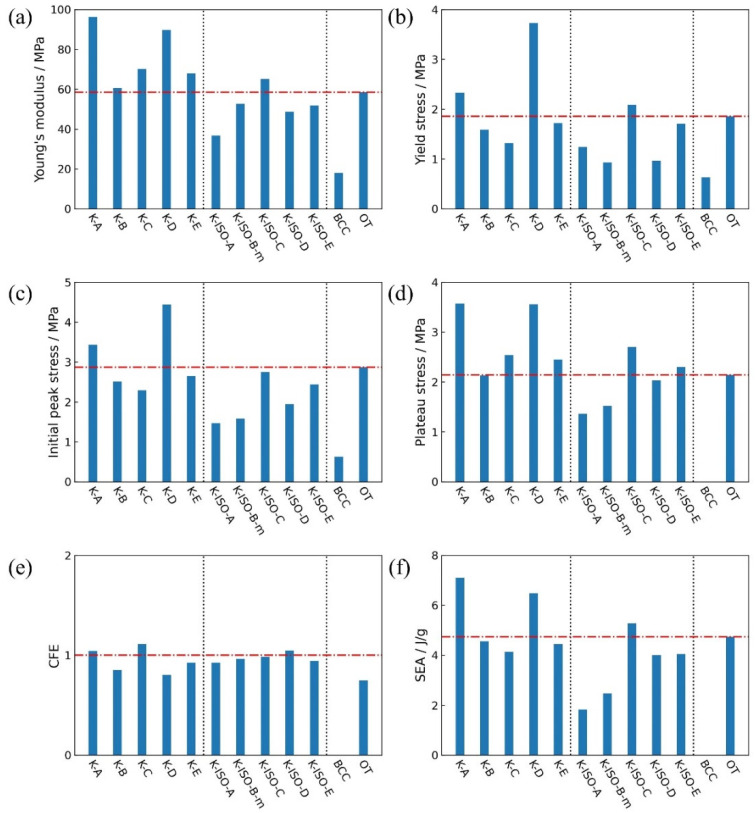
Quasi-static mechanical properties of different lattice structures (The dotted lines represent different types of lattice structures; for (**a**–**d**,**f**), red dashed line represents the mechanical properties value of OT; for (**e**), red dashed line represents CFE = 1.): (**a**) Young’s modulus; (**b**) yield stress; (**c**) initial peak stress; (**d**) plateau stress; (**e**) impact force efficiency; (**f**) specific energy absorption.

In this section, the Young’s modulus, yield stress, initial peak stress, plateau stress, crushing force efficiency (CFE), and specific energy absorption (SEA) of the above optimized lattices, as well as OT and BCC lattices, are compared and discussed, as shown in Figure 18. It is worth noting that some indexes of the BCC lattice are blank, as the initial densification strain is required to calculate them. However, the nominal stress–strain curve of the BCC structure has not reached the densification stage. It can be observed that all the mechanical properties of BCC are the lowest. Figure 18a shows that the Young’s modulus of the topology-optimized lattice structures with cubic symmetry is higher than that of OT. As for the Young’s modulus of topology-optimized lattice structures with isotropic constraint, the K-ISO-C is higher than that of OT, while the modulus of the remaining configurations is slightly lower than that of OT. Figure 18b indicates that the yield stress of the K-A, K-D, and K-ISO-C is higher than that of OT, while Figure 18c shows that only the initial peak stress of the K-A and K-D is higher than that of OT. Figure 18d indicates that the plateau stress of K-A, K-C, K-D, K-E, K-ISO-C, and K-ISO-E is higher than that of OT. The CFE of K-A and K-ISO-C is the closest to 1, as exhibited in Figure 18e, which implies that the linear segment and the plateau segment on the nominal stress–strain curves of these two lattice structures are relatively stable. Figure 18f presents that the specific energy absorption of K-A, K-D, and K-ISO-C is higher than that of OT. It demonstrates that the energy absorption capacity of these three lattice structures is better than that of OT under quasi-static compression in the current loading direction. The above results confirm that novel lattice structures with superior mechanical properties can be designed by topology optimization.

### 4.4. Direction Dependence of Topology-Optimized Lattice Structures

**Figure 19:**
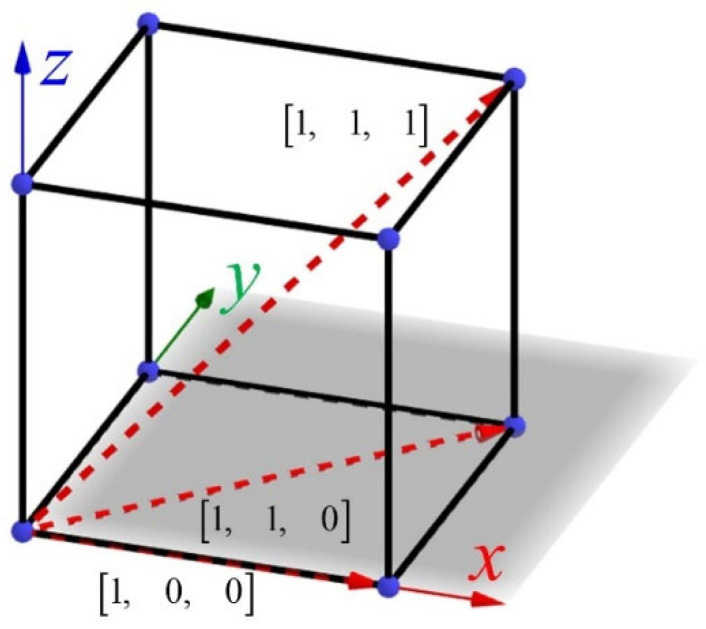
Three main directions of lattice structure.

The anisotropy of the lattice structure can be evaluated by the elastic modulus of the lattice structure in the three principal axis directions. In this section, the mechanical properties of the lattice structure in the three principal axis directions are studied. K-A and K-ISO-C lattices are the representatives of the two types of topology-optimized structures. The direction dependence regarding the mechanical properties of the two topology-optimized lattice structures is investigated by the finite element method (FEM). The three principal directions of the lattice structure can be expressed as [1, 0, 0], [1, 1, 0] and [1, 1, 1] by Miller indices, as shown in Figure 19.

**Figure 20:**
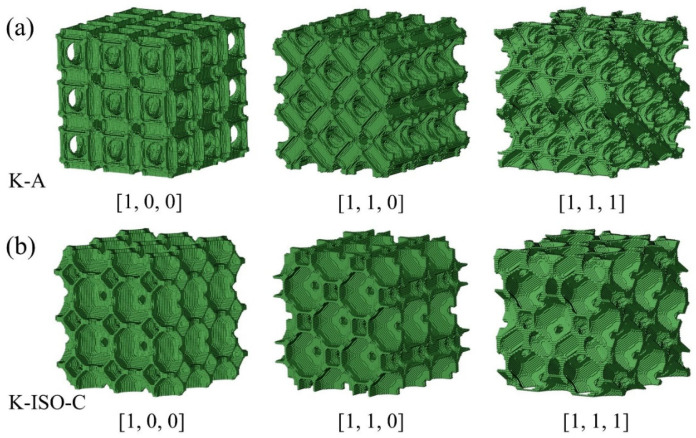
Topology configurations of two lattice structures in three principal directions: (**a**) K-A lattice structure, (**b**) K-ISO-C lattice structure.

Figure 20 exhibits the configurations of K-A and K-ISO-C when loaded in three principal directions. These configurations were imported into Abaqus for numerical calculation. It can be captured from Section 4.2 that when the strain exceeds 0.3, the configurations begin to fracture. For simplicity, only the mechanical behavior of the lattice structures before failure (ε ≤ 0.3) is predicted in the numerical simulations.

**Figure 21:**
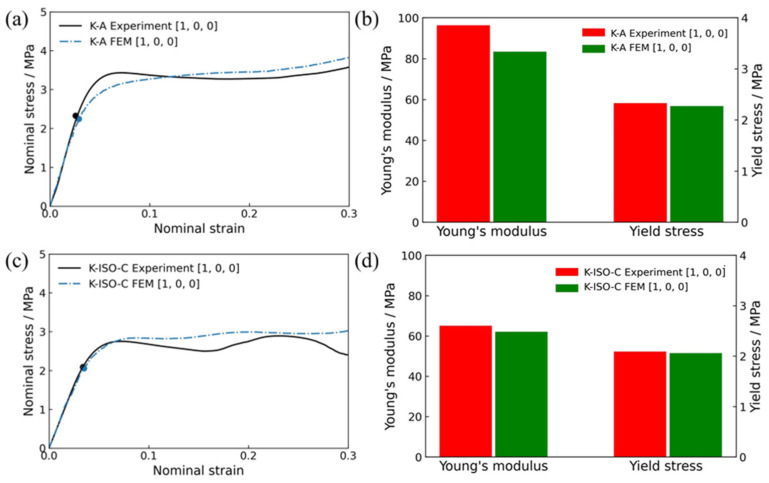
Mechanical behavior of two lattice structures under compression along [1, 0, 0] direction: (**a**) experimental and numerical stress–strain curves of K-A lattice structure, (**b**) Young’s modulus and yield stress of K-A lattice structure, (**c**) experimental and numerical stress–strain curves of K-ISO-C lattice structure, (**d**) Young’s modulus and yield stress of K-ISO-C lattice structure.

K-A represents the maximizing bulk modulus considering volume constraints, achieving the theoretical upper bound of bulk modulus. K-ISO-C represents the maximizing bulk modulus considering volume and isotropic constraints. It can balance high bulk modulus and isotropy well among the listed samples. Therefore, K-A and K-ISO-C have been selected as the representative structures in this section. Figure 21 presents the mechanical properties of K-A and K-ISO-C under quasi-static compression along the [1, 0, 0] direction. Among them, Figure 21a and c are the comparison diagrams between the experimental and numerical stress–strain curves of the two lattice structures. It can be found that the nominal stress–strain curves of the two lattice structures predicted by numerical simulation are basically consistent with the experimental results in the elastic and stress plateau stages (Figure 21a, R^2^ = 0.984; Figure 21c, R^2^ = 0.957). Figure 21b,d is the Young’s modulus and yield stress of the two lattice structures obtained by numerical simulations. For K-A, the difference between Young’s modulus obtained by simulation and experiment is 13.19%, and the difference in yield stress is 2.58%. For K-ISO-C, the difference in Young’s modulus is 4.69% and in yield stress is 1.44%. The above results show that the numerical models can effectively and accurately determine the mechanical behavior of lattice structures subjected to quasi-static loading.

**Figure 22:**
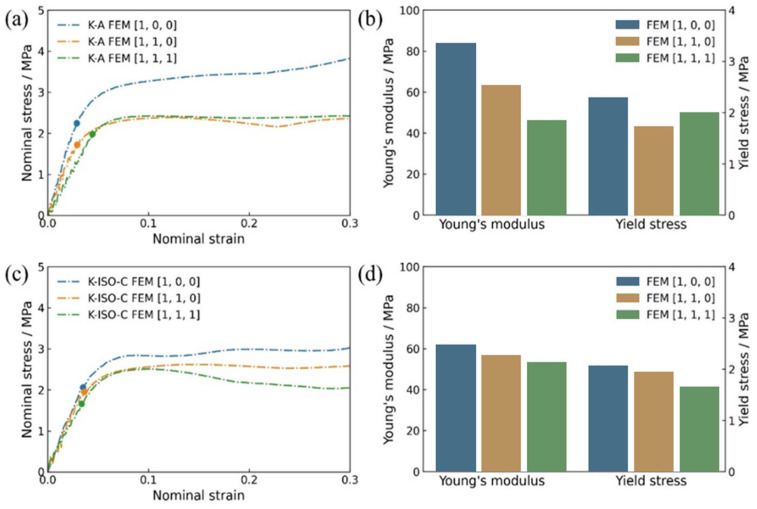
Mechanical properties of the two lattice structures under quasi-static loading in three principal directions: (**a**) nominal stress–strain curves of K-A lattice structure, (**b**) Young’s modulus and yield stress of K-A lattice structure, (**c**) nominal stress–strain curve of K-ISO-C lattice structure, (**d**) Young’s modulus and yield stress of K-ISO-C lattice structure.

The mechanical properties of the two lattice structures in three principal directions under quasi-static loading obtained by numerical simulations are presented in Figure 22. The solid dots on the curves in the figure represent the yield stress of the lattice structure in the corresponding loading direction. Figure 22a shows the nominal stress–strain curves of the K-A lattice structure in three principal directions. It can be observed that the elastic stage of K-A exhibits obvious direction dependence. The Young’s modulus of K-A in the [1, 0, 0] direction is the highest, while in the [1, 1, 1] direction is the lowest, as displayed in Figure 22b.

Additionally, taking the mechanical properties of the lattice structure in the [1, 0, 0] direction as a reference, the differences in mechanical properties in different directions are calculated and discussed by ($V_{\left[1,0,0\right]}\;-\;V_{\min}$)/$V_{\left[1,0,0\right]}$ × 100%. Here, *V_min_* denotes the minimum value regarding different mechanical parameters. The maximum difference between Young’s modulus in different directions is 44.40%, and it is 24.23% for yield stress. To characterize the dependence direction of stress in the nonlinear stage, the mean stress $\sigma_{\mathrm{mean}}$ is defined as

(28) $$\sigma_{mean}=\frac{\int_{\varepsilon_{y}}^{0.3}\sigma \left(\varepsilon \right)d\varepsilon }{0.3-\varepsilon_{y}}$$

where $\varepsilon_{y}$ is the strain corresponding to the yield stress. The average stresses of K-A in three directions are 3.36 MPa, 2.27 MPa, and 2.38 MPa, respectively. Among them, the maximum deviation between the average stresses is 32.44%. Figure 22c shows the nominal stress–strain curves of K-ISO-C in the three directions. Although a certain difference can be observed on the curves of K-ISO-C in the elastic stage, the dependence on the direction has been significantly reduced compared with K-A. Moreover, the largest Young’s modulus of K-ISO-C is in the [1, 0, 0] direction and the smallest in the [1, 1, 1] direction. The Young’s modulus and yield stress of K-ISO-C determined from the simulation results are exhibited in Figure 22d. The mechanical properties in the [1, 0, 0] direction are also used as a reference to evaluate the difference between the mechanical properties of K-ISO-C in different directions. The largest difference between Young’s modulus in different directions is 14.42%, which is 19.42% for yield stress. The average stresses in the three directions of K-ISO-C are 2.86 MPa, 2.53 MPa, and 2.24 MPa, respectively. The maximum difference between the mean stresses is 21.68%. The above results demonstrate that the mechanical properties of K-A with cubic symmetry present obvious direction dependence. However, the direction dependence of K-ISO-C optimized with isotropic constraints has been significantly reduced. It should be noted that although only the elastically isotropic constraint is considered during topology optimization, the nonlinear mechanical properties (such as yield stress and mean stress) of K-ISO-C are also significantly less dependent on direction than K-A with only cubic symmetry.

## 5. Conclusions

In this work, topology-optimized lattice structures with elastic isotropy and maximum bulk modulus are designed based on BESO. The superiority of the optimized lattice structures has been validated through numerical simulations and experiments compared with traditional lattice structures. The main conclusions are as follows:

(1) The anisotropy index of topology-optimized lattice structures with elastically isotropic constraint is around 1. The selection of filter radius in topology optimization presents a significant influence on the generation of lattice structures. By controlling the filter radius, different configurations of lattice structures can be designed to meet various functional requirements.

(2) When the volume fraction is 0.2, the topology-optimized lattice structures exhibit superior mechanical properties over traditional OT and BCC lattices. Meanwhile, the experimental results reveal that K-A lattice structures exhibit a relatively homogeneous deformation mode without local collapse under quasi-static compression, which results in their more excellent loading capacity. The K-ISO-C structure exhibits uniform coordinated deformation, thereby ensuring stability under multidirectional loading.

(3) Unlike lattice structures that possess only cubic symmetry, the finite element analysis confirms that the elastic modulus of topology-optimized lattice structures with an elastically isotropic constraint is almost independent of the loading direction. Furthermore, both the yield stress and the average stress of isotropic lattice structures are less sensitive to the loading directions than those of cubic symmetric lattice structures, according to the FEA result. The topology-optimized lattice structures demonstrate significant potential for industrial applications requiring complex load-bearing capabilities. Specifically in biomedical implants, aerospace components, and energy absorption structures.

## Figures and Tables

**Table 1:** Size and mass of additive manufacturing lattice structures.

Topology	Number	Measured Dimensions (mm)	Mass (g)
K-A	1	36.78 × 36.37 × 36.36	12.20
	2	36.75 × 36.37 × 36.40	12.19
K-B	1	36.22 × 36.64 × 36.17	12.16
	2	36.24 × 36.50 × 36.15	12.16
K-C	1	36.55 × 36.50 × 36.49	12.03
	2	36.32 × 35.47 × 36.20	12.07
K-D	1	36.34 × 36.34 × 36.33	12.05
	2	36.30 × 36.34 × 36.26	12.12
K-E	1	36.26 × 36.51 × 36.26	12.19
	2	36.26 × 36.43 × 36.17	12.14
K-ISO-A	1	36.65 × 36.76 × 36.28	12.15
	2	36.66 × 36.78 × 36.18	12.08
K-ISO-B-m	1	36.40 × 36.56 × 36.19	12.10
	2	36.47 × 36.52 × 36.28	12.07
K-ISO-C	1	36.30 × 36.42 × 36.09	12.18
	2	36.41 × 36.50 × 36.12	12.12
K-ISO-D	1	36.23 × 36.37 × 36.05	12.10
	2	36.30 × 36.37 × 36.12	12.11
K-ISO-E	1	36.18 × 36.34 × 36.18	11.97
	2	36.21 × 36.36 × 36.20	12.01
OT	1	36.15 × 36.19 × 36.14	12.01
	2	36.19 × 36.36 × 36.17	12.05
BCC	1	36.30 × 36.37 × 36.22	11.80
	2	36.24 × 36.29 × 36.24	11.95

## Data Availability

The original contributions presented in the study are included in the article, further inquiries can be directed to the corresponding authors.

## Abbreviations

The following abbreviations are used in this manuscript:

BESO	Bidirectional evolutionary structural optimization
SLA	Stereo Lithography Appearance
OT	Octet-truss
BCC	Body-centered cubic
RVE	Representative volume element
CFE	Crushing force efficiency
SEA	Specific energy absorption
FEM	Finite element method
